# The commercialization of traditional medicine in modern Cambodia

**DOI:** 10.1093/heapol/czx144

**Published:** 2017-10-11

**Authors:** Bandeth Ros, Gillian Lê, Barbara McPake, Suzanne Fustukian

**Affiliations:** 1 ReBUILD Consortium and Health Research Unit, Cambodia Development Resource Institute, 56, St 315, Phnom Penh, PO Box 622, Phnom Penh, Cambodia; 2 Health Systems Governance and Finance Unit, Nossal Institute for Global Health, University of Melbourne, Victoria, 3010, Melbourne, Australia; 3 Nossal Institute for Global Health, University of Melbourne, Victoria, 3010, Melbourne, Australia, ReBUILD Consortium, and Queen Margaret University, Musselburgh, EH21 6UU Edinburgh, UK; 4 ReBUILD Consortium and the Institute for Global Health and Development, Queen Margaret University, Musselburgh, EH21 6UU Edinburgh, UK

**Keywords:** Traditional medicine, commercialization, affordability, UHC, Cambodia

## Abstract

Globally, traditional medicine has long been used to address relatively common illness, mental ill health and during childbirth and post-natal care. However, traditional medicine is primarily provided by the private sector and it is unclear how far expenditures on traditional medicine contribute to household impoverishment. A life history method was used to understand the health seeking experience of 24 households over the last 60 years in Cambodia, a country with high out-of-pocket expenditures for health. The life histories suggest that traditional medicine in Cambodia has been undergoing a process of commercialization, with significant impacts on poor households. In the earlier lives of respondents, payments for traditional medicine were reported to have been flexible, voluntary or appropriate to patients’ financial means. In contrast, contemporary practitioners appear to seek immediate cash payments that have frequently led to considerable debt and asset sales by traditional medicine users. Given traditional medicine‘s popularity as a source of treatment in Cambodia and its potential to contribute to household impoverishment, we suggest that it needs to be included in a national conversation about achieving Universal Health Coverage in the country.

Key Messages

Traditional medical practitioners in Cambodia have expanded their markets—treating patients from a larger geographic catchment and providing a wider range of services that now include aspects of biomedicine.

Traditional medicine in Cambodia has been undergoing a process of commercialization, with significant impacts on poor households.

Given its potentials to contribute to household impoverishment, policy makers need to include traditional medicine in the national conversation about achieving Universal Health Coverage (UHC) in the country.

## Introduction

Traditional medicine is an important component of healthcare for a large proportion of the global population. Several estimates have put global use at about 80% of total population (e.g. Tovey *et al.* 2007; Chawla *et al.* 2013; Chege *et al.* 2015; Aziato and Antwi 2016). At the same time, traditional medicine is overwhelmingly provided in the private sector. In countries with a dominant commercialized private sector, recurrent use of the private sector for health care correlates with high levels of out-of-pocket payments (OOPs) capable of inducing or maintaining poverty. Financial protection against such negative impacts is an important component of universal health coverage (UHC) that now sits at the heart of the 2030 Sustainable Development Goal for Health and Wellbeing.^1^ As a private sector phenomenon that is commonly accessed across the socioeconomic status range, the contribution of traditional medicine to out-of-pocket expenditure is potentially important. We examine financing of traditional medicine in a country with one of the highest levels of out-of-pocket healthcare expenditures as percentage of total health expenditure in the world—Cambodia (WHO 2013).

Global use of traditional medicine is hard to estimate but it appears that the use of traditional medicine is ubiquitous. ‘Traditional Medicine’ is a category that covers a wide range of medical traditions in low- and middle-income countries and tends to be labelled ‘complementary’ or ‘alternative’ medicine in high-income countries (e.g. Bodeker and Kronenberg 2002). While it was earlier supposed that traditional medicine was cheap in LMICs when compared with biomedicine (e.g. Agbor and Naidoo 2011), more recent fieldwork has shown that herbal treatments, at least, can be more expensive. The cost of herbal remedies in Kenya, for example, was ‘prohibitive’ by Kenyan standards of living, while such remedies were not covered by insurers and so could only be financed out-of-pocket (Chege *et al.* 2015). Similar evidence was found in Vietnam (Dalgetty 2010) and Cameroon, Tanzania and Ethiopia (Leonard 2000). In Tanzania, diviners also charged high prices, substantially higher than those associated with biomedicine (Muela *et al.* 2000).

Traditional medicine is used by all wealth quintiles, though poorer people use traditional medicine more often and face a higher relative burden of out-of-pocket expenditure from its use. Leonard (2000) argues this is ‘almost entirely due to a geographic effect’ in which those living in remote areas, who are mostly poor, must pay for additional travel costs, a finding supported in Cameroon where poor patients were willing to travel long distances to meet with a desired practitioner (Labhardt *et al.* 2010). Families of epileptic children in Cameroon chose traditional medicine even though it was 20 times more expensive than biomedicine (Njamnshi *et al.* 2009a, b; see also Muela *et al.* 2000). In South Africa, use of traditional medicine is associated with being employed since those who have a regular income are in a better position to meet such costs (Sorsdahl *et al.* 2009). One agreed reason for its popularity is that traditional medicine is significantly more patient-centred in several aspects (Sorsdahl *et al.* 2009; Labhardt *et al.* 2010; Merriam and Muhamad 2014).

Outside of some Asian countries (e.g. China, India, Japan, South Korea, Vietnam), traditional medicine is generally provided in the private sector. It can account for a significant share of total household healthcare treatment episodes—49% in one Bangladesh study (Rahman *et al.* 2013). This implies that traditional medicine could potentially account for a high share of household expenditure on healthcare as financial protection measures for service users of this sector usually do not exist. Accessing care in the private sector is not solely a function of cost, however. Financial affordability for the household, considering direct and indirect costs, is important, but ‘interplays’ (McLaughlin and Wyszewianski 2002) with two other factors. The first is physical accessibility of services, such as geographic reach and other modes of service organization and delivery that allow people to obtain services when they need them. The second is acceptability, or the willingness of people to seek and take up services on offer (Evans *et al.* 2013). The interplay of these factors produces observable patterns of health service use and healthcare financing decisions by households (Levesque *et al.* 2013) within a dynamic environment of public and private service supply. However, within this, the use and impact of traditional medicine on household expenditures is consistently overlooked. This article contributes to filling that gap, taking a longitudinal approach to show how the contribution of traditional medicine to household impoverishment is a relatively recent phenomenon in Cambodia.

In Cambodia, the 2014 Demographic and Health Survey notes that 1.5% of the population sought traditional medicine treatments (Kru Khmer/monk) in the month prior to the survey taking place (NIS et al. 2015). This likely underestimates the use of traditional medicine over a longer period such as a year, as the DHS captures only the events of the last 30 days before the survey. The Ministry of Health (MOH) estimates of 40–50% of the population using traditional medicine (WHO and MOH 2012) is more consistent with global averages. Private expenditure on health as a percentage of total expenditure on health in Cambodia stood at 78% in 2014 (WHO 2014). A high proportion of OOP was spent on private care—both formal and informal, and expenditures on traditional medicine were classed as ‘informal’ within the National Health Accounts from which these data derive (OECD *et al.* 2011). Use of savings (31.3%), borrowing money (12.4%) and selling assets (7.6%) were all common coping strategies to pay these health costs, alongside using wages/earnings (64%) (NIS et al. 2015). The private health sector in Cambodia mainly performs curative services and is composed of licensed and unlicensed providers. Cambodia has about 5500 licensed private providers that are mainly located in urban areas (WHO 2015). Given known problems with licensing and registration systems in LMICs (Ensor and Weinzierl 2007), it is likely that the real number of private practices is much higher.

Cambodia’s national policy on traditional medicine was adopted in 2010. It aimed for safe, effective and quality products and practices through the promotion of ethical practice, provision of training, encouraging rational use and integration with biomedicine, and regulation of production and distribution of products (MOH 2010). However, traditional medicine is not yet included in the National Health Sector Strategic Plan nor covered by health insurance (WHO and MOH 2012). The total number of practitioners is not known, although it is thought that at least one practitioner resides in every village and several in larger villages (WHO and MOH 2012). The sector is lightly regulated—since 1998, practitioners are required to obtain a certificate and gain government approval to practice, but it is unclear what proportion of practitioners conforms with this regulation. The National Centre of Traditional Medicine provides a short course-training programme but it is unclear how systematically training is taken up and the required approvals obtained. The sector is diverse with numerous practices and treatments. For clarity, a typology of practices proposed by Guillou (2009) is set out in Table 1. This is not an exhaustive list of traditional medicine treatments in Cambodia as there are overlaps between these practices, and some individual practitioners may perform multiple roles and treatments across this typology. Our respondent’ accounts sometimes described experiences contradicting the categories such as monks whose services were not provided free of charge. Most of these types of practice were found during the research that underpins this article but a number of different names, not included in the table were used. It is outside the scope of this article to follow the shifting definitions and categories of traditional practitioners and we cite the labels used exactly rather than attempt to map them to any fixed categorization system.

**Table 1.** Typology of traditional practitioners (Guillou 2009)

Name used	Type of therapy	Source of legitimacy	Socioeconomic group of therapist	Socioeconomic group served	Type of competence	Specialty	Remuneration
Lay Nonn	Neo-traditional *Qrū*	Government/ traditional	Civil servant/ business person	Upper	Acquired knowledge	Physical illness	Fixed price
Chea Sophon							
						Mental illness	
Kong Kaev	Traditional *Qrū*	Traditional	Peasant	Low	Acquired knowledge; handed-down knowledge	Physical illness; Mental illness;	Free service or flexible price or gift
						Management of events;	
						Prediction	
Lam Hong	Monk	Religious	Peasant	Medium	Acquired knowledge; handed-down knowledge	Physical illness; Mental illness;	Free service
						Management of events;	
						Prediction	
Penh Yon	Spirit medium	Traditional	Peasant	Low	Handed-down knowledge	Physical illness; Mental illness;	Free service
						Management of events;	
						Prediction	
	Masseurs	None (marginalized)	Peasant	Low	Acquired skill	Physical illness	Fixed price
						Prostitution	

Source: translated from Guillou (2009)

Having provided a brief contextual background, we next describe the methods underpinning the research and go on to trace changes in uptake and affordability of traditional medicine in Cambodia over the lifetimes of older Cambodian heads of households.

## Methods

**Figure 1.**
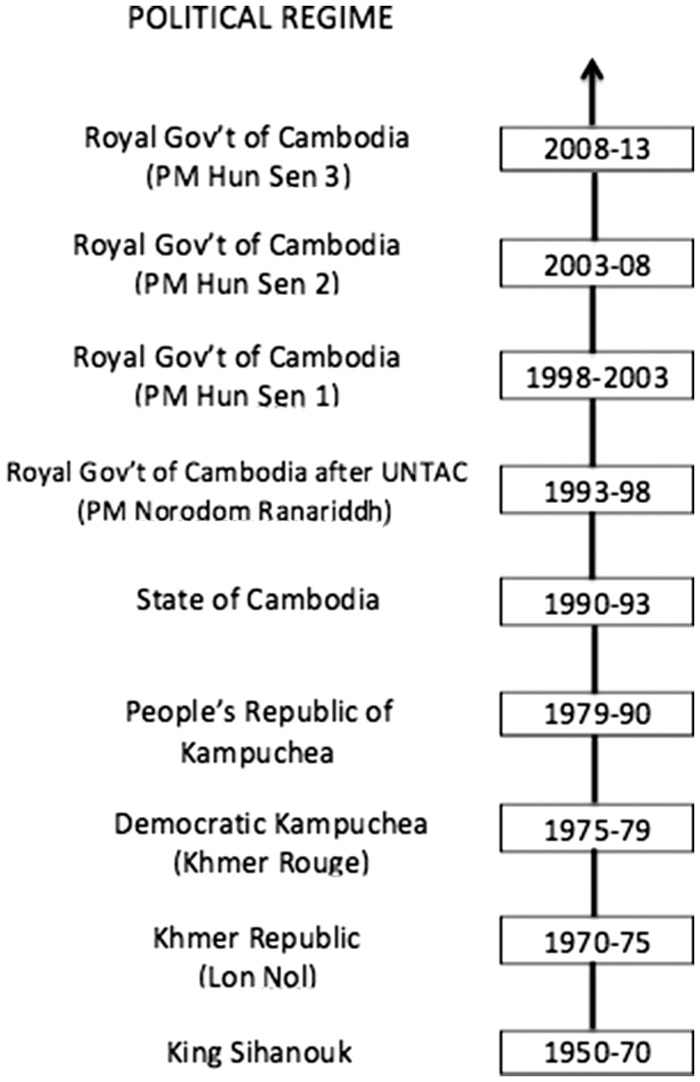
Political Regime in Cambodia from 1950 to 2013

The data for this article were generated through a study that focused on health-seeking behaviour and catastrophic spending faced by Cambodian people across different political regimes from the latter half of the twentieth century. The regimes mentioned are set out in a timeline below for clarity (see Figure 1).

A qualitative approach was employed using life histories to collect information on episodes of illnesses, deaths and births from respondents through time and their health seeking behaviour. For this article, the interview data focused on household use of traditional medicine were extracted and discussed. The interview guides covered the following topics:

Episodes of major illness in life or the family

- What was the illness?
- When and who suffered it?
- What did you do?
- Did you choose traditional medicine?
- Why did you do that?
- How did you decide where to go?

Payments

- What did you have to pay?
- Could you afford it?
- Did you get any help?
- How did you manage to pay?
- What did you do, if you couldn’t manage?

The study was carried out in 2013 in four Operational Health Districts (ODs) in Phnom Penh (Sen Sok, West, North and South) and two in Takeo province (Ang Roka and Kirivong) reflecting urban and rural locations. These six ODs were also characterized by high-poverty levels and had long experience in operating health equity and insurance funds. A total of 24 interviewees were purposively recruited using the following criteria. First, poor and non-poor people were interviewed. Poor households were identified through discussion with village chiefs, local health NGO operators and cross-checking to a list of persons who were already identified as poor by the national ID-Poor government programme. Non-poor households were selected with assistance of local health NGO operators among their clients who used health insurance scheme or general households, who experienced several health treatments and difficulties in coping with health spending. Second, so that sufficient life stories over time could be collected, only heads of households, male or female, who were 40 years old or older were recruited. The wider objectives of the study were to explore the health seeking pathways followed by Cambodians, identify the impacts of health expenditure incurred and how the household coped over time from the pre-conflict to post-conflict periods. For this reason, the age of the respondent was particularly important and it was considered that Heads of Household were most likely to have an overview of the experience across household members. The number of interviews reflected time and budget constraints. The interviewer screened participants prior to interview to ensure that they had sufficient memory of health treatments. Ethics approval was received from both the National Ethics Committee for Health Research in Cambodia and Queen Margaret University in the UK. Informed consent was gained for all interviews.

Interviews were conducted, recorded and transcribed first in Khmer (the main language of both locations and of Cambodia) and thereafter translated into English. Table 2 shows the responses from the 24 life histories, of which 23 indicated they sought traditional treatment. At the time of the interview, the householders, 6 male and 18 female ranging in age from 40 to 74, were living in urban (Phnom Penh districts) or rural settings (Takeo districts) (although this was not necessarily their residence at the time of traditional medicine use).

**Table 2.** Interviewees

Age	Gender	Location	Poverty category	Number of stories	Periods of stories	Treatment sought
74	M	Urban	Poor	3	1967; 1984; 2012–2013	Herbalist; Buddhist monk
68	M	Urban	Non-Poor	2	1955; 1993	Herbalist
67	M	Rural	Non-Poor	2	1950-–1970; 2013	Herbalist
63	F	Rural	Non-Poor	1	1973	Spirit medium;
63	F	Rural	Poor	6	1968; 1969; 1970; 1971; 1989; 2013	Herbalist; black magic
62	F	Rural	Non-Poor	1	1980	Herbalist
60	F	Urban	Poor	2	1972; 1989	Herbalist
59	F	Urban	Non-Poor	3	1961; 1975; 1993–1994	Herbalist
57	M	Rural	Non-Poor	1	1995	Black magic
57	F	Rural	Non-Poor	6	1976; 1977; 1978; 1979; 1996; 1997–1998	Herbalist
55	F	Rural	Non-Poor	3	1986; 2010–2012; 2013	Herbalist; Qrū sa’nṭhit; spirit medium
54	M	Rural	Non-Poor	3	2005; 2010; 2013	Herbalist
53	F	Urban	Non-Poor	2	1970–1975; 1984	Herbalist;
52	F	Urban	Poor	1	2011	Bone setting
51	M	Urban	Poor	4	1973; 1984; 2003; 2009	Herbalist; black magic; spirit medium; bone setting
47	F	Urban	Non-Poor	1	2000–2010	Herbalist; Buddhist monk
46	F	Urban	Non-Poor	1	1975–1994	Herbalist; spirit medium
46	F	Urban	Poor	3	1974–1975; 1986; 2005	Herbalist
45	F	Rural	Poor	1	1992	Herbalist
45	F	Urban	Poor	–	–	No mention of TM
44	F	Rural	Poor	1	2010	Herbalist
41	F	Urban	Non-Poor	1	1979–2013	Herbalist
41	F	Rural	Poor	7	1984; 1991; 1992; 1993; 1998; 1999; 2012	Herbalist
40	F	Rural	Poor	2	1975–1979; 1997	Herbalist; *Qrū sa’nṭhit*; spirit medium

Our analysis indicated that use of traditional birth attendants (TBA) in the current era had become rare for antenatal, birth and post-birth care, as the inverse of much increased health facility use for such care across the whole population. Therefore, we did not consider TBA to be a major source of impoverishment from traditional medicine use and removed it from further analysis.

A coding framework was used to process the interview data, structured in chronological order, as follows: types of illness; service use, specifically type of service sought; location; when sought; and cost. Transcripts were reviewed and excerpts inserted into the framework, then compared across time lines and themes. Reflections were shared between co-authors through several stages to confirm, challenge, and feedback into further refinement of the themes presented here.

We recognize five potential limitations of our analysis. The data were obtained from 2 out of 25 provinces—the findings may reflect regional rather than national trends. Further, the data source for this article was interviews and is therefore subject to reliance on memory recall. The total number of interviews is small (24), meaning that we can use the data for insight but we cannot assess traditional medicine coverage or the incidence of its associated household expenditure on healthcare expenditure nationally or regionally. In addition, although the sample was designed to contrast urban, rural, poor and non-poor, these categories were unstable over the life course and after analysis, current status did not predict patterns in the responses. We have therefore pooled the data for analysis. Last, as an artefact of the study‘s wider objectives, all interviewees were over 40 years old—it may be that the older population is more willing to use traditional medicine than younger people, and there are likely to be other structured differences between their experiences and those of younger people.

## Findings

Three themes became apparent in the interview data: the pattern of resort for traditional medicine has changed over time; geographical reach of traditional medicine practitioners has increased; and the methods and levels of payment have changed. We present our findings by theme, using interview excerpts. First, however, we present one summarized life history to give a flavour of the dataset to readers.

## A life history

Śīthā (pseudonym) lived in Phnom Penh and was 74 years old at the time of the study. He could detail his care-seeking behaviours since the French colonial period. He started his story by telling how senior villagers were often the ones to perform traditional treatment for their community: ‘*The past time was not like today… a grandfather named Tā Vung treated us for illness that looked like diarrhoea and dengue fever, with some herbs and leaves that he picked up on the wayside. He mixed and squeezed these into a drink… then prayed. It was effective. I believe in our traditional herbs medicine, and that is why we [Cambodian people] still continue to use it until today.*’ In the King Sihanouk period, Śīthā reported the common use of traditional medicine because of poor accessibility to public facilities: ‘*We did not really go to hospital for just normal fever unless we fainted… some people still did not go to hospital and died. It was too far, and it is not like today where we have referral hospital or commune health centers… . It wasn’t like today where we have a lot of medicine available.*’ In the Khmer Republic period, Śīthā’s first child had measles and he sought treatment from an herbalist: ‘*I went to a Qrū Thnāṃ. In the past, we believed that when having disease like measles we just go to a Qrū … . and he soaked leaves and tree branches and we bathed in the water. We did not trust in medical doctors.*’ During the Democratic Kampuchea (Khmer Rouge period), Śīthā’s fourth child died from an illness called ‘Bis '´āc kuk’, but he did not mention the use of traditional medicine to intervene in the illness episode. Rather he talked about having a villager who was assigned by the government to treat illness—this person had no medical training of any kind. During the Vietnamese occupation, Śīthā experienced hepatitis and sought treatment at a government hospital in Phnom Penh and was hospitalized for almost 3 months until his recovery. He later suffered hepatitis three times (in 2010, 2012 and 2013). The first two times he went to a different government hospital in Phnom Penh, but for the third episode he switched to traditional medicine: ‘*The medical doctors advised me not to drink alcohol. They said if I drink alcohol, the sickness would reappear and I might not be able to survive. After a while, I had the disease again. I have been recommended to go to the same hospital again, but I felt ashamed that I did not follow the doctor’s advice so I decided not to go to the hospital. I went to several traditional medicine practitioners before I met one that could effectively cure my disease. I gave [the first practitioner] a small sum of money to show my gratitude. But, I have spent a lot with the Kán Ḍiang herbalist (the second practitioner) around 300–400 US dollars.*^2^*I was in severe condition that my family even thought that I wouldn’t have survived for long. Luckily, the Buddhist monk [the third practitioner] came to help me on time. His treatment was really effective …but I spent around 1000 US dollars [for his offerings].*’

## Changing pattern of resort for traditional medicine

In common with Śīthā‘*s* story, most life histories showed that Cambodians still use traditional medicine today, despite a wider variety of biomedical public and private health care services available in the country. However, there has been a change in how and when they are used. From the French colonial period to the present day, traditional medicine was perceived as an appropriate treatment for relatively common illnesses (such as measles), broken bones, childbirth and post-natal care or mental illness.

*‘A: I thought that [bone] fracture could be treated by traditional medicine and take less time than at hospital. The pain disappeared after one week, and she spent another month to recover completely and go back to work…*’ (M, 51, Phnom Penh).

In the past, traditional medicine was automatically the first line of resort for an illness. However, in current times, there is a clear change to using traditional medicine when illnesses are considered untreatable by biomedicine or when there are other reasons for rejecting available biomedical practitioners, such as concerns about interpersonal treatment, and biomedicine becomes unacceptable. Traditional medicine is still used but seems to be changing to a second line of resort.

‘*All doctors in (the public) referral hospital, Takeo provincial town and Phnom Penh didn’t know what kind of disease my daughter had—sometimes she laughed for no reason, and sometimes she knew me and sometimes she didn’t. It was really difficult for me.*’ (F, 55, Takeo).

*‘A: He fell from this house and broke his arm. I first sent him to the health center, but then he was referred to the Municipal Hospital. The doctor scolded my husband…My husband got furious…After the argument, my husband and I brought the grandson back home. My husband tried to look for a bone setting practitioner to cure the broken arm.*’ (F, 52, Phnom Penh).

Cambodians have tended to combine the services of both traditional and biomedicine since the end of the Democratic Kampuchea period. Across all time periods, interviewees were quick to switch between medical traditions if they found either to be ineffective or unacceptable.

*‘Q: She already had medicines from the hospital, why she needed to go for traditional medicine? A: Because we were worried about her and wanted her to get better as soon as possible. She took both medicines and traditional medicine at same time.*’ (F, 55, Takeo).

## Increased geographical reach of services

Traditional medicine practitioners lived within or nearby the communities they served during the time of King Sihanouk or the Khmer Republic. In these periods, practitioners and clients either directly knew or knew of each other through village or family networks.

*‘Q: How did you get to know the herbalists? A: I knew them because they lived in my village. They were specialized in the field, but this time they couldn’t help my child*’ (F, 63, Takeo)

In the State of Cambodia regime and after the United Nations Transitional Authority in Cambodia UNTAC (1992–1993), five interviewees reported that they started looking for traditional medicine practitioners from further afield, following advice from friends or relatives, but still within the province.

‘*Q: How many Qrū sa’nṭhit*^3^*did you take him to? A: There were three Qrū sa’nṭhit, and all of them said that my husband was cursed. Q: Where was the first Qrū sa’nṭhit? A: He was at Trábāṃng jvia. The second one was at south of Trábāṃng '´áṇḍoek, and the third one was at north of Tual thlung. Q: How do you know those Qrū sa’nṭhit? A: I heard it through word of mouth.*’ (F, 40, Takeo)

During the 2000s, interviewees often reported travelling to different provinces. This was possible because newly constructed roads or improvements to existing roads made it faster for motorbikes to move. In addition, marketing and public media (radio) were now used. One interviewee, whose treatment by biomedicine had failed, responded to a claim heard on the radio about a cure for chronic or severe illnesses.

‘*I heard the announcement from radio about Khmer traditional medicine that it could cure such disease. I bought it for 300 US dollars and my daughter felt better after taking it.*’ (M, 54, Takeo).

‘*I took a mototaxi and went alone. I didn’t want to bother any of them [adult children]. They had things to do. I spent 5 US dollars for mototaxi to travel from my house to Uḍung pagoda (in Kampong Speu province) to see a Buddhist monk and back.*’ (M, 74, Phnom Penh)

Another finding that emerged was the changing relationship between client and the traditional medicine practitioner. Such relationships were formerly personal but, over time, this had changed to something more commercial. In the past, practitioners and clients worked together to identify the cause of the illness through negotiation and dialogue. After receiving treatment and recovering, the client was expected to acknowledge and respect the work of the practitioner by providing gifts to show respect. Since the early 2000s, interviewees reported that the treatment process had become shorter. While some practitioners still required a certain period of time for treatment, others restricted the time spent with clients focusing more on the sale of herbal medicine. Interviewees complained of a lack of empathy for the client‘s distress. One practitioner refused to continue the treatment.

‘*It was a boil, which had not come to a head, so the practitioner had his method to prevent it from spreading and cure it. He applied medicines on it. I gave him 3 US dollars for each time he came to cure my son by spewing magical water on the boil. However, it was such a waste because my son got even worse. Later, he refused to come to spew again because he said that the boil was beyond his treatment capacity. He asked me to take my son to the hospital.*’ (F, 46, Phnom Penh)

‘*Q: And how long did you stay with the Kán diang herbalist? A: I just went to buy the medicine from him… I have taken a lot of medicine—and it was really hopeless until I met the last practitioner…*’ (M, 74, Phnom Penh)

From the King Sihanouk period to that of the Royal Government of Cambodia, interviewees referred to traditional medicine and biomedicine as separate bodies of knowledge with separate treatment methods. For instance, herbalists used herbs, leaves or dead wild animals to cure illnesses, while biomedical doctors used intravenous drips, injection, modern medicine, blood tests or other diagnosis and surgery methods. In the current period, however, there appears to be the dual provision/merging of traditional medicine and biomedical practices. One interviewee, for example, discussed how the herbalist they visited used biomedical evidence to support and complement his treatment, which raised the cost of care.

‘*Q: The herbalist asked you to go for blood test? A: Yes, once receiving the x-ray result, I had to bring it to the herbalist so that he could diagnose hepatitis A, B or C, and then, he would prescribe the medicine for me. Q: How much did you spend on x-ray and echo test? A: I had to go for various tests many times, not just once. Q: Do you remember how often you went for the tests? A: I just went for the tests until I lost all my trust on the healer and went to see the new herbalist. I have spent a lot with the Kán diang herbalist. Around two or three thousand US dollars. And I had to go for x-ray. I just went to buy the medicine from him. Actually, the medicine is not expensive, only around 5 US dollars, but I had to spend a lot on x-ray.*’ (M, 74, Phnom Penh).

## Changed methods of payment

Various expenditures were reported. In the past, interviewees claimed that traditional medicine practitioners did not demand fees and that payments were voluntary. Rather, gifts were made based on what the client had to hand:

‘*Q: Did the herbalist who treated your eye disease ask you for money? A: He did not ask for money, just a handful of bananas [the usual ritual offerings]. He did not ask for any money, and I just gave him any available offerings I could afford such as bananas or just little money. He said that he did not take the offerings for himself, but for his master [a particular type of personal spirit called Qrū.*’ (F, 46, Phnom Penh).

Interviewees reported that traditional medicine practitioners also provided unpaid services, such as sharing a recipe or ingredients to make up an herbal remedy, or advice on methods of treatment to use at home. However, this practice was becoming less common and clients were now being asked to pay. We also found reports that traditional medicine has become more expensive, with demands for high treatment fees that required the client to sell assets to pay them, leaving them at risk of slipping into poverty.

‘*A: I tried with the herbalist for three months, but was not cured. I spent more than 270 US dollars on that and sold one cow. I bought a packet of the traditional medicine every 10 days that cost 7 US dollars. I soaked that medicine in 2.5 litres of alcohol and drank it for 10 days and then I soaked another pack in 2.5 litres and drank it again.*’ (F, 44, Takeo).

‘*Q: How much have you spent on the treatment by the last herbalist so far? A: Around 200 US dollars. He charges for the medicine and his traveling fee when he came to visit me which includes gasoline and a meal. He said he would charge only 2 US dollars for a pack of medicine if I can go to his house directly.*’ (M, 74, Phnom Penh)

Traditional medicine users also slipped into poverty when they moved between different types of traditional medicine practice, each one requiring consultation fees, treatment costs and transportation. Four interviewees reported taking out a loan or requesting additional financial assistance from relatives to continue to meet these costs. Paying back the debt was the responsibility of the client and all family members living under the same roof. For instance, we found one man who called on his adult children to contribute to the cost of treatment he had received from an herbalist, while supporting their own families at the same time.

*‘Q: How about your children? Are they very helpful to you when you are sick? A: They helped me with the money to their capability to pay back to the bank and support their own family. They work from dawn to dusk and also have to think about their own children.*’ (M, 74, Phnom Penh).

In the end, six interviewees ran out of cash to pay the debt and sold an asset that would have been used for their on-going livelihood. One interviewee described the loss of cattle to pay for her husband‘s treatments. Since then, she has had no cattle for ploughing.

*‘Q: How can you have that money? A: It came from my saving money from selling wood. Did you sell anything else for his treatment? A: I sold another cow during his treatment and a plot of rice field. At that time the land was cheap. Now, I don’t have any cows left.*’ (F, 63, Takeo).

## Discussion

Cambodians report using traditional medicine throughout their lifespans. It has stood the test of time (a point made by Sato and Costa-i-Font 2012 in the context of Africa) and seems likely to persist in the future. Many traditional medicine practitioners belong to the category of informal private provision, but even with increased affordability and competence of care in the public sector provision of biomedicine since the late 1990s, informal private practice of traditional medicine persists.

Traditional medicine has been, and continues to be, resorted to for a wide range of serious and non-serious illness. However, our interviews appear to show a trend that traditional medicine is now being resorted to when biomedicine provided in the public sector cannot provide a cure, or the trust of service users in public sector biomedical doctors is absent. This has also been found in Gryseels *et al.* (2013) (traditional medicine was resorted to for malaria when the patient did not feel cured with antimalarial tablets), and Eisenbruch (1994) (traditional medicine was resorted to for some symptoms of mental illness when the formal health system failed to provide care). When families and patients turned away from biomedicine in the public sector they resorted to traditional medicine in the private sector, and not to biomedicine in the private sector. We did not ask interviewees the reason for this but suggest that, with dual practice common, it is likely that private biomedicine is provided by the same medical staff at a higher cost. A larger survey would be required to confirm this shift in pattern of resort for traditional medicine to second line, when biomedicine is seen to fail.

In more recent times, the interviews suggest that traditional medical practitioners have expanded their markets—treating patients from a larger geographic catchment and providing a wider range of services that now include aspects of biomedicine. This expansion has capitalized on improved transport and communications within the country. Since the mid-1990s, mass media infrastructure such as TV, radio and the internet have all contributed to improved access to a wider array of information; the construction and improvement of roads has enabled travel, internally and into neighbouring countries. Using such communication channels is not unique to Cambodia and has been documented elsewhere. For example, a study in Ghana showed that traditional medicine practitioners advertise on the radio, newspaper, TV and internet (Aziato and Antwi 2016); and in a Malaysian study, three practitioners had their own internet page and had been featured in local TV programmes, newspapers and magazines (Merriam and Muhamad 2014). Such studies show that while they may be called ‘traditional’, many practitioners are increasingly familiar with contemporary marketing practices and building a client base. In the future, their services may have a national not just local market, and add considerable pluralism to local health markets.

Payments have also changed. In the past, these were flexible, voluntary or appropriate to the patients’ own financial means. They are now mainly cash single payments or multiple instalments that have led to debt and asset sale by service users in some instances. This contrasts with other countries. A Cameroon survey showed that patients made higher payments when cured and lower when not, and frequently included a symbolic gift. The fixed payment often varied by illness and the payment period could be negotiated (e.g. up to 7 years in one case) (Leonard 2000). In Malaysia, traditional medicine practitioners ‘usually take the socioeconomic background of individual patients into consideration’ (Merriam and Muhamad 2014). With the benefit of longitudinal research, we can observe that this used to be the case in Cambodia (see also Collins 2000) but has waned in recent years. With increased use and increasingly national coverage, it is likely we will see accelerating expenditures in traditional medicine and an increased proportion of impoverishment (out of all healthcare related impoverishment) related to its use.

Based on our data, we suggest that traditional medicine in Cambodia has been undergoing a process of commercialization, with significant impacts on poor households. After the Khmer Rouge genocide, the interviewees in our study increasingly reported fees being charged rather than ‘gifts’, and the word disappears altogether between the 1990s and 2010. At the same time, fees seem to be increasing and interviewees showed an awareness of being exploited on occasion. Trankell and Ovesen (2007) argue that the loss of many of the spiritual aspects of care from traditional medicine practices and significant written knowledge during the Khmer Rouge regime has ‘impoverished’ traditional practices—in terms of spirituality and the empathy generated throughout the care seeking episode. Ultimately this means ‘the poor have reduced access to… treatment they would otherwise be able to afford economically’ (ibid p. 58). An overall interpretation of the interview data is that privately provided traditional medicine becomes highly acceptable, leading to its users’ overcoming physical and financial constraints, when publicly provided biomedicine becomes inaccessible or unacceptable. If this is indeed the case, a portion of private traditional medicine users from poor households become highly vulnerable as financial protection for direct costs of care and indirect costs (e.g. transportation) is not available.

Traditional medicine is still relevant to households in contemporary Cambodia. Poor households have persistently used traditional medicine during times when public services were limited, and later, when such services did become available. Our data show that elderly Cambodian poor households have long experience of using traditional medicine; that it is trusted; and perceived as a relevant healthcare provider in Cambodia’s contemporary plural health market. Further research is needed to understand the scale of traditional medicine use, confirm the pattern of resort indicated in the interview data, and understand whether patterns of traditional medicine use differ by age group or socioeconomic background. However, given the continuing relevance of traditional medicine to the poor, it is important for policy makers to acknowledge the fact and include traditional medicine in the national conversation on how UHC will be achieved. Traditional medicine is often overlooked in health policy, considered a footnote to the achievement of public health goals, and a diminishing one at that. Given its sheer popularity and its potential to contribute to household impoverishment, UHC will not be achieved if traditional medicine is left out in the cold.

## Funding

This article was based on the ReBUILD Consortium work funded by UK Aid, Department for International Development. All views expressed here are those of the authors alone.

## Footnotes

1 United Nations http://www.un.org/sustainabledevelopment/health/, accessed 29 July 2016.

2 Participants sometimes describe expenditures in Cambodian Riels and sometimes in US dollars which are used interchangeably. We have translated all riel expenditures into US dollars at approximately (not all accounts are specific about the year they occurred) historical exchange rates (from the historical rates quoted by xe.com) and current (2017) values based on http://www.usinflationcalculator.com/, and we have rounded them to the nearest dollar, hundred dollar or thousand dollar amount.

3
*Qrū sa’nṭhit* ‘stay with’ refers to a healer who can connect one or more spirits such as guardian spirits to help them diagnose causes of illness and disease.
